# Prediction of Micro-Milling-Induced Residual Stress and Deformation in Titanium Alloy Thin-Walled Components and Multi-Objective Collaborative Optimization

**DOI:** 10.3390/ma19020219

**Published:** 2026-01-06

**Authors:** Jie Yi, Rui Wang, Dengyun Du, Dong Han, Xinyao Wang, Junfeng Xiang

**Affiliations:** 1 School of Mechanical and Electronic Engineering, Shandong Jianzhu University, Jinan 250101, China; 2 School of Technician Department, Shandong Labor Vocational and Technical College, Jinan 250300, China; 3 School of Civil Aviation, Northwestern Polytechnical University, Xi’an 710072, China; 4 Yangtze River Delta Research Institute of NPU, Suzhou 215400, China; 5 Innovation Center NPU Chongqing, Northwestern Polytechnical University, Chongqing 401135, China

**Keywords:** micro-milling, residual stress, deformation prediction, GA-BP neural network, multi-objective optimization

## Abstract

The intrinsically low stiffness of titanium alloy thin-walled components causes residual stresses to readily accumulate during high-speed micro-milling, leading to deformation and hindering machining precision. To clarify the residual-stress formation mechanism and enable deformation control, this study first proposes a surface residual stress characterization model based on an exponentially decaying sinusoidal function, with model parameters efficiently identified via an improved particle swarm optimization algorithm, allowing rapid characterization of stress distributions under different process conditions. A response surface model constructed using a central composite design is then employed to reveal the coupled effects of machining parameters on residual stress and top-surface deformation. On this basis, a GA-BP neural network–based prediction framework is developed to improve the accuracy of residual stress and deformation prediction, while the AGE-MOEA2 multi-objective evolutionary algorithm is used to optimize micro-milling parameters for the simultaneous minimization of residual stress and deformation via Pareto-optimal solutions. Validation experiments on thin-wall micro-milling confirm that the optimized parameters significantly reduce peak residual stress and suppress top-surface deformation. The proposed modeling and optimization strategy provides an effective reference for high-precision machining of titanium alloy thin-walled components.

## 1. Introduction

The rapid development of the aerospace industry has imposed increasingly stringent requirements on product structural design, functionality, and performance, with lightweight and integrally machined configurations emerging as a predominant trend (Jiang et al. [1]; Zhan et al. [2]). This trend has driven the widespread adoption of low-density light alloys, thin-walled structures, and large-scale integrated components, which offer superior performance but face inherent challenges in machining. Due to their small wall thickness and low structural rigidity, thin-walled parts are prone to significant dimensional deviation after the release of clamping forces, primarily attributed to the elastic recovery of residual stresses. Compared with conventional parts, residual stress exerts a more pronounced influence on low-stiffness thin-walled components-even moderate or low levels of residual stress can induce substantial secondary deformation (Ding et al. [3]). This not only affects machining quality and workpiece mechanical performance (Ding et al. [3]) but also poses challenges for subsequent processing and service stability.

Regarding the generation mechanism of milling-induced residual stress, several studies have elaborated on it from a microscale perspective: beyond stress formation, phase transformation also plays a role in residual stress evolution, as shown by Mahdi and Zhang [4], who found that surface hardening and volumetric expansion from phase transformation shift residual stress from compressive to tensile in EN23 alloy steel. It can be observed that the severe material deformation coupled with cutting heat during the milling process collectively contributes to the generation of residual stress.

Building on this foundation, several works have conducted relevant experiments to investigate the main factors influencing the thermo-mechanical coupling effect on residual stress; for instance, Mohammadpour et al. [5] reported that increased cutting speed and feed rate elevate surface tensile residual stress in AISI 1045, while Sasahara et al. [6] highlighted that multiple tool passes alter residual stress distribution by changing cutting forces or shear angles. Caruso et al. [7] examined the interactive effects of tool-edge geometry, workpiece hardness, cutting speed, and microstructural evolution on residual stress in AISI 52,100 steel, and Ventura et al. [8] found that tool designs increasing flank face-workpiece contact length enhance subsurface compressive residual stress in AISI 5115 steel. Capello et al. [9] further refined these insights, identifying tool nose radius and feed rate as the primary factors influencing surface residual stress in C45 and 39NiCrMo3 steels, with cutting speed and rake angle exerting only minor effects. Zawada Michałowska [10] reported that residual stress is strongly dependent on cutting speed, with its variation trend closely mirroring the observed pattern of cutting force changes. These works have systematically confirmed that cutting parameters such as milling speed and feed rate, as well as different tool geometries, exert significant influences on residual stress. Furthermore, to further reveal the formation mechanism of residual stress, several studies have established finite element models to analyze the characteristics of milling-induced residual stress. For example, Miguélez et al. [11] demonstrated through finite element simulations that mechanical action alone—driven by the interplay of plastic bulging and burnishing effects—can generate surface tensile residual stress in the absence of thermal effects. Finite element models, including those by Nasr [12], Ulutan [13], and Özel and Ulutan et al. [14,15], provide detailed stress field simulations. Fuh and Wu [16] employed a response surface approach combined with the Taguchi method to predict milling-induced residual stress in 2014-T6 aluminum alloy, with the mathematical model accounting for both cutting parameters and tool geometry. Lazoglu et al. [17] proposed an enhanced analytical model that superimposes mechanical and thermal stresses before relaxation, while Wang et al. [18] developed a model tailored to peripheral milling of TC4 titanium alloy. Sun et al. [19] reconstructed a three-dimensional residual stress model by incorporating feed rate and milled surface residual area. Jia et al. [20] integrated finite element simulations with orthogonal experiments to capture residual stress and cutting force distributions. Agrawal et al. [21] adopted a similar approach to predict residual stress in orthogonal cutting of AISI 4340 steel and validated the results experimentally. Yi et al. [22] developed a data-driven quantitative model to reveal the mechanisms by which multiple milling parameters affect machining accuracy, applying an improved NSGA-III algorithm for dynamic optimization to obtain a set of non-dominated solutions, followed by entropy-weighted TOPSIS for visualization and determination of the unique optimal solution. The aforementioned studies have explored the formation mechanism and key influencing factors of residual stress from the perspectives of microscale mechanisms, experiments, and simulations. Building on this foundation, to more accurately characterize the properties of residual stress and deformation, several studies have conducted relevant quantitative analyses from a mathematical analytical perspective. For example, Fergani et al. [23] developed a physical model for grinding temperature based on Timoshenko’s thermoelastic theory, identifying grinding temperature as the dominant factor inducing tensile residual stress with minimal mechanical stress contribution. Yi et al. [24] established deformation equations for micro-thin wall structures with mixed boundaries under concentrated milling forces, providing insights into the deformation mechanisms of thin-walled components. Empirical models, such as those proposed by Saini et al. [25] using Box–Behnken design response surface methodology and Mittal and Liu [26] via regression techniques, offer practicality for specific process conditions. Xiang et al. [27] established analytical models for surface morphology and residual stress after milling and ultrasonic surface rolling. Xiang et al. [28] investigated dynamic milling force control strategies to improve thin-walled component machining accuracy. Yi et al. [29] further reviewed milling chatter in aerospace thin-walled structures, covering dynamic characterization techniques that support comprehensive process optimization.

Building on the exploration of the key influencing factors and analytical perspectives of residual stress mentioned above, several studies have further shifted toward process optimization to achieve active control of residual stress and machining quality. For example, Sembiring et al. [30] used artificial neural networks to predict residual stress and hardness of nickel-based alloys after UNSM treatment. Li, Ren et al. [31,32] reported that optimal cutting depth maintains low surface residual stress to facilitate deformation control, while Zhang et al. [33] modeled longitudinal rolling residual stress of aluminum bars without addressing multi-objective tradeoffs. Jafarian et al. [34] optimized only the tensile direction of residual stress in Inconel 718 turning, and Dabade [35] used grey relational analysis to improve surface integrity of Al/SiCp composites by optimizing key parameters. Other studies have explored error compensation (Soori et al. [36]), stress relief (Wang et al. [37]), and the impact of initial residual stress (Schulze et al. [38]), while Yue et al. [39] employed the NSGA-III algorithm to optimize surface microhardness considering residual stress relaxation, representing a step toward multi-objective optimization. Chen et al. [40] combined grey relational analysis, BP neural networks, and NSGA-III to identify residual stress-influencing factors and establish predictive models. Kartheek [41] optimized Inconel 718 milling to minimize surface roughness and residual stress while maximizing material removal rate. Yi et al. [42,43] advanced optimization frameworks—including RVEA-entropy weight TOPSIS and improved NSGA-III—and established micro-milling force models accounting for dual flexibility coupling deformation, providing robust tools for process optimization.

Therefore, to clarify the mechanisms of residual stress and deformation in micro-milling titanium-alloy thin-walled components, this study develops an exponentially decaying sinusoidal model solved by an improved PSO to obtain stress distribution characteristics under different parameters. A CCD-based response surface model is then established to reveal the effects of key machining variables. A GA-BP neural network is introduced to improve prediction accuracy, and the AGE-MOEA2 algorithm is applied for multi-objective optimization of micro-milling parameters. Validation experiments confirm that the optimized parameters effectively reduce residual stress and control deformation.

## 2. Empirical Modeling and Prediction of Residual Stress Distribution in Milled Surface Layers

### 2.1. Curve Model for Residual Stress Distribution

**Figure 1:**
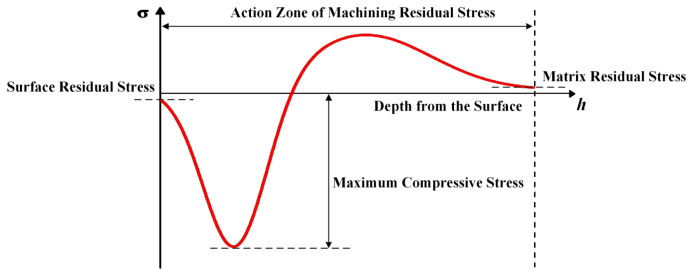
Residual stress distribution curve.

The distribution of residual stress along the depth direction of the machined surface typically exhibits a bowl-shaped profile, as illustrated in Figure 1. This behavior arises because mechanical extrusion and plastic deformation induced by the cutting tool dominate in the outermost layer. After being extruded and elongated, the surface material is constrained by the underlying elastic substrate, resulting in a compressive stress zone. As thermal effects become significant during cutting, the surface layer experiences tensile stress upon cooling and contraction, leading to the formation of a tensile stress peak. With increasing depth, the thermo-mechanical influence imposed by machining gradually diminishes, and the residual stress eventually returns to its initial state in the bulk material. Such a distribution can be effectively characterized using a curve-based model.

In this study, an exponentially decaying sinusoidal function is employed to characterize the residual stress profile, incorporating both an exponential attenuation term and its corresponding sinusoidal oscillation term, as expressed in Formula (1). Compared with traditional fitting approaches, the proposed formulation more accurately reflects the finite-dimension conditions of actual workpieces. During model identification, an *R*^2^ constraint is imposed to ensure fitting accuracy. A larger *R*^2^ value indicates a higher degree of goodness-of-fit, as given in Formula (2).

(1) $$\sigma (h)=\zeta \cdot (1-h/H)\cdot e^{-\lambda \kappa h/\sqrt{1-\lambda^{2}}}\sin(\kappa h+\varphi )$$

where ζ is a constant, λ and κ denote the attenuation coefficient and the oscillation frequency, respectively; φ is the phase angle, *h* represents the depth of the stress measurement point, and *H* is the total depth.

(2) $$\max\left[R^{2}=1-\frac{\sum {\left(y_{i}-y_{p}\right)}^{2}}{\sum {\left(y_{p}-\bar{y_{p}}\right)}^{2}}\right]$$

where $y_{i}$ denotes the fitted value, $y_{p}$ represents the measured value, and $\bar{y_{p}}$ is the mean of the measured values.

### 2.2. Model Solution Using an Improved Particle Swarm Optimization Algorithm

Due to the inherent drawbacks of the conventional Particle Swarm Optimization (PSO) algorithm—such as slow convergence and a tendency to fall into local optima—this study introduces an improved PSO framework. The enhanced algorithm accelerates convergence while improving stability, effectively balancing global exploration and local exploitation capabilities. The magnitude of the inertia weight *ω* directly affects the particles’ search dynamics; it regulates the trade-off between exploration and exploitation by determining the degree to which previous velocities influence subsequent updates. To address this, a nonlinear dynamic inertia-weight adjustment strategy is proposed. This approach enables broad global exploration during the early iterations and facilitates precise convergence toward the global optimum in later stages, as expressed in Formula (3).

(3) $$\omega (t)=\omega_{\max}{\left(\frac{\omega_{\min}}{\omega_{\max}}\right)}^{\left(1-\frac{t}{t_{\max}}\right)}$$

where $\omega_{\max}$ and $\omega_{\min}$ denote the maximum and minimum inertia-weight coefficients, respectively, t and $t_{\max}$ represent the current iteration number and the maximum number of iterations.

The learning factors regulate the cognitive abilities of both individual particles and the swarm. When the learning factors take relatively large values, particles tend to search rapidly outside the target region, thereby expanding the overall search space, but at the risk of missing the global optimum. In contrast, when the learning factors are small, particles focus their search within a narrow region around the target, which restricts the exploration range and makes it difficult for the particles to escape local optima. In this study, an asynchronous learning-factor strategy is proposed, wherein a logarithmic function is introduced to construct nonlinear, asynchronously varying learning factors. This design enhances the balance between global exploration and local exploitation within the algorithm, as expressed in Formulas (4) and (5).

(4) $$c_{1}=c_{\max}-(c_{\max}-c_{\min})\ln\left(1+(e+1)\frac{t_{\max}-t}{t_{\max}}\right)$$

(5) $$c_{2}=c_{\min}+(c_{\max}-c_{\min})\ln\left(1+(e+1)\frac{t_{\max}-t}{t_{\max}}\right)$$

where $c_{\max}$ and $c_{\min}$ denote the upper and lower bounds of the learning factors.

**Figure 2:**
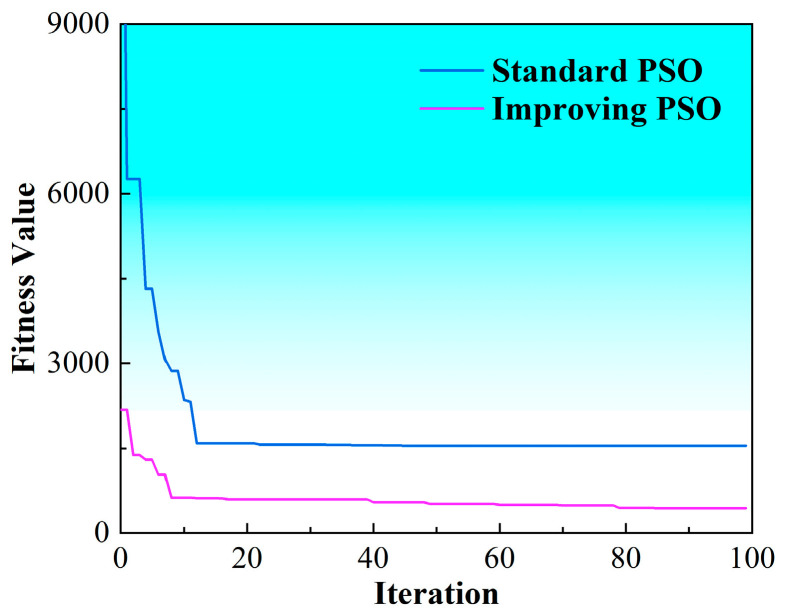
Convergence comparison of the algorithm curves.

The comparison of fitness–iteration evolution curves before and after the algorithmic enhancement is presented in Figure 2. The analysis indicates that the improved PSO algorithm exhibits a markedly faster convergence rate relative to the standard PSO formulation. Moreover, it is capable of escaping local optima at earlier stages of the search process and demonstrates substantially enhanced global exploration capability.

### 2.3. Establishment of the Residual Stress Distribution Curve Representation Model

In this study, a two-factor, three-level orthogonal experimental design was formulated, with feed per tooth (*f*_z_) and cutting speed (*v*_c_) selected as the key influential factors. This experimental design yielded nine test conditions, and the corresponding data-including the cutting parameters, the distance of the measurement point from the machined surface (*h*), and the corresponding residual stress value (*σ*)-are summarized in Table 1. Based on these data, a representation model for the surface residual stress distribution curve was constructed and solved using the improved particle swarm optimization algorithm.

**Figure 3:**
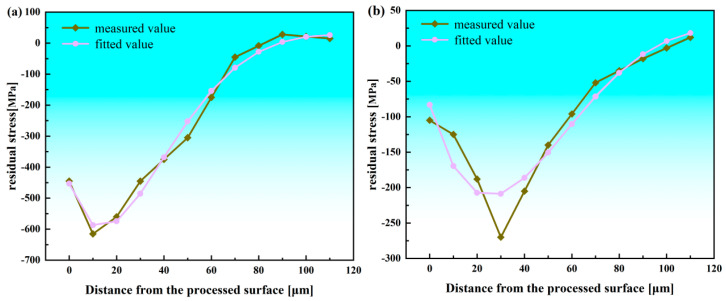
Characterization of surface residual stress distribution. (**a**) Best-fitting curve; (**b**) worst-fitting curve.

The characterization coefficients of the residual stress curves are summarized in Table 2. As shown, the coefficient of determination *R^2^* ranges from 0.91 to 0.98, indicating that the proposed residual stress curve representation model achieves high fitting accuracy and effectively captures the typical features of the residual stress distribution. The best and worst-fitting curves are illustrated in Figure 3.

**Figure 4:**
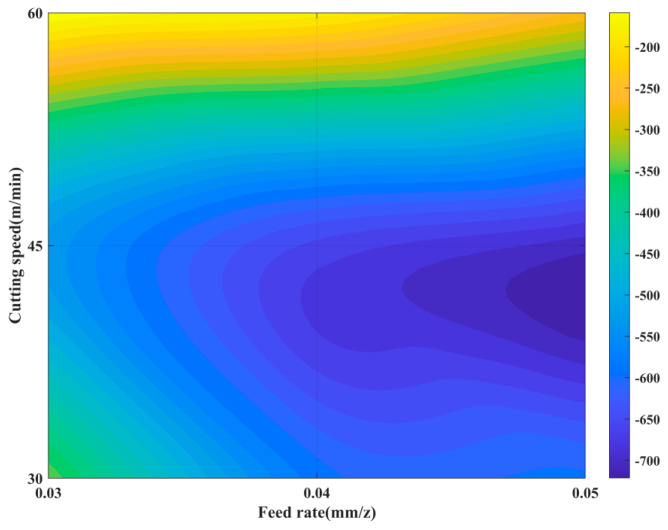
Response contour map of compressive stress extrema.

Using the constructed residual stress curve prediction model, the extreme compressive stress of the curves was identified as a key parameter, and the dynamic evolution of extreme compressive stress with respect to cutting speed *v*_c_ and feed rate *f*_z_ was generated, as shown in Figure 4. The model predictions indicate that the extreme compressive stress in the surface residual stress field exhibits a significant positive correlation with feed rate, whereas the effect of cutting speed is nonlinear: the compressive stress initially increases with rising cutting speed, reaching a critical value before subsequently decreasing. These results demonstrate that the proposed prediction model can accurately and effectively characterize the distribution features of residual stress within the defined parameter space.

## 3. Experimental Investigation of Thin-Walled Part Micro-Milling

### 3.1. Design of the Micro-Milling Process Scheme

**Figure 5:**
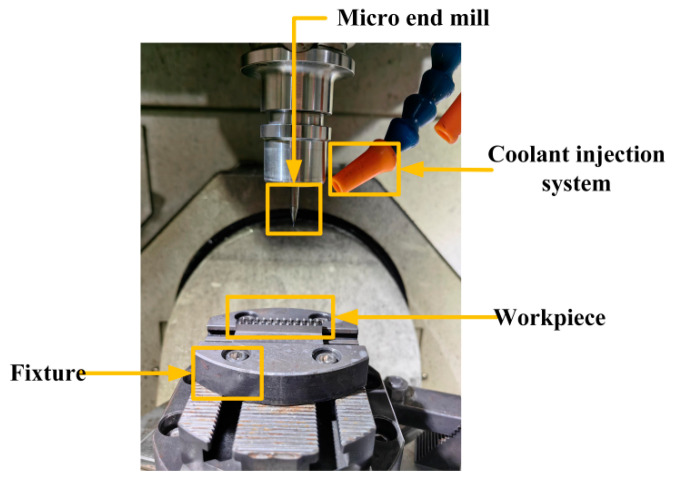
Micro-milling device.

Micro-milling experiments were conducted to investigate the influence of key process parameters on machining accuracy, with particular focus on the top-surface deformation and dimensional integrity of the curved thin-walled structure. The experiments were carried out on a GF Mikron HSM500 three-axis high-speed machining center with a maximum spindle speed of 24,000 r/min, as shown in Figure 5. A four-flute NS MHRH430R end mill was employed, featuring a diameter of 0.5 mm.

The workpiece material was TC4 titanium alloy, and each specimen measured 40 mm × 10 mm × 5 mm. A curved thin-walled structure with a thickness of 80 μm and a height of 900 μm was machined into each specimen, comprising 12 thin-walled units per sample. In this study, residual stress (*σ*) and top-surface deformation (*d*) were selected as the response variables. The top-surface deformation reflects the overall geometric deviation of the curved micro–thin wall and serves as a key indicator for evaluating dimensional accuracy in thin-wall micro-milling. Residual stress, on the other hand, directly affects the service stability of thin-walled components as well as subsequent machining processes.

To establish the quantitative relationships between the machining parameters and the response variables, a response surface model was constructed using a central composite design (CCD). Four micro-milling parameters were selected as the design variables, each assigned three levels. The specific experimental configuration is presented in Table 3, and the corresponding response values were recorded accordingly.

**Figure 6:**
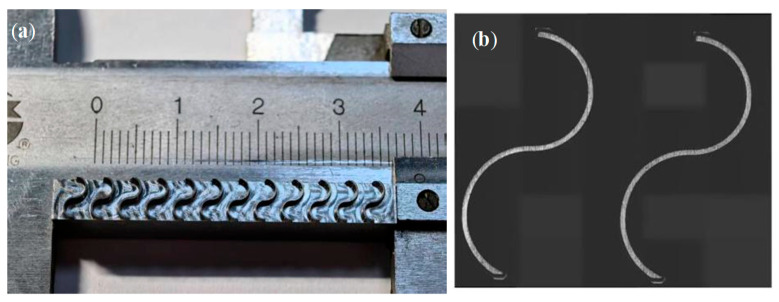
Finishing of curved thin-wall structure. (**a**) Machined structure; (**b**) structure design diagram.

**Figure 7:**
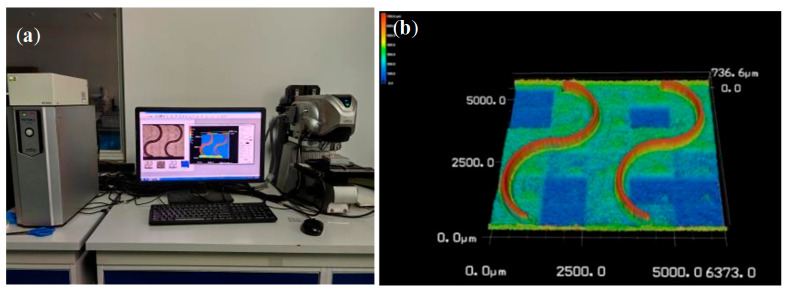
(**a**) Top surface profile thickness measurement; (**b**) 3D morphology of curved thin wall.

**Figure 8:**
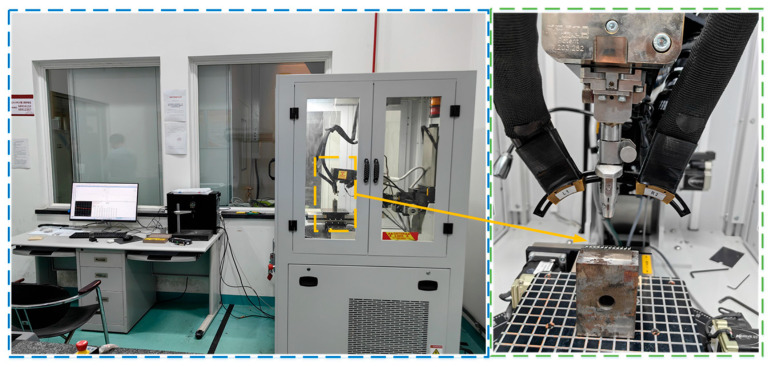
Residual stress measurement device.

The machined workpieces and their corresponding design are shown in Figure 6. The top-surface geometric morphology was measured using a Keyence VK-X200K laser confocal microscope, which provides a height resolution of 0.001 µm. Figure 7 presents the sampling locations for the top-surface profile thickness after machining, as well as the 3D morphology of the curved thin wall. Residual stress was measured using an LXRD X-ray diffraction residual stress analyzer. The workpiece was subjected to layer-by-layer corrosion and stripping using an electrolytic polishing machine with a saturated NaCl electrolyte solution. Residual stress in the shallow surface layer was measured at five equally spaced points on the inner side of the top surface of the curved micro-thin wall, employing a Cu-target X-ray tube with a diffraction angle of 139.3°. The response value was recorded as the average of three residual stress measurements, and the measuring equipment is illustrated in Figure 8.

### 3.2. Analysis of Experimental Results and Model Validation

A second-order polynomial was employed to fit the response surfaces and to compare the design points with their corresponding predicted values. This approach establishes functional relationships between residual stress, top-surface deformation, and cutting parameters for TC4 titanium alloy under high-speed micro-milling conditions. The resulting quadratic response surface model is expressed in Formula (6).

(6) $$Y=\beta_{0}+\sum \beta_{i}x_{i}+\sum \beta_{ii}x_{i}^{2}+\sum \sum \beta_{ij}x_{i}x_{j}+\varepsilon$$

where Y denotes the response; $\beta_{i}$, $\beta_{0}$ represents the regression coefficients; $x_{i}$ is the factor under investigation; and ε is the estimation error. To avoid model overfitting and enhance prediction robustness, stepwise regression analysis was adopted for variable selection in the initial full-factorial second-order polynomial model. Terms with statistically insignificant effects (*p* > 0.05) were eliminated. The final simplified models (Formulas (7) and (8)) only retain the linear terms, quadratic terms, and interaction terms that exert significant influences on the response indicators. The fitted mathematical relationships among σ, d and the variables *n*, *f_z_*, *a_e_*, *a_p_* are given in Formulas (7) and (8).

(7) $$\begin{array}{l} \sigma =77.653290+0.001904\times n+12.490800\times f_{z}+0.748178\times a_{e}-0.504648\times a_{p}\\ -0.000466\times nf_{z}+0.000029na_{e}-6.031330\times 10^{-6}\times na_{p}+0.071772\times f_{z}a_{e}\\ +0.016938\times f_{z}a_{p}+0.001181\times a_{e}a_{p}+2.104350\times 10^{-7}\times n^{2}-0.961471\times {f_{z}}^{2}\\ -0.017337\times {a_{e}}^{2}+0.000861\times {a_{p}}^{2} \end{array}$$

(8) $$\begin{array}{l} d=90.7800-1.2200\times n+2.2300\times f_{z}+0.2843\times a_{e}+1.3500\times a_{p}-0.4522\times nf_{z}\\ +0.8653na_{e}+0.0125\times na_{p}-1.0600\times f_{z}a_{e}-0.9562\times f_{z}a_{p}-0.0861\times a_{e}a_{p}\\ +1.9000\times n^{2}-0.0644\times {f_{z}}^{2}+0.6451\times {a_{e}}^{2}-0.6252\times {a_{p}}^{2} \end{array}$$

The analysis of variance (ANOVA) indicates that the overall model exhibits a *p* value of less than 0.0001, demonstrating that the model can accurately characterize the complex relationships between residual stress, top-surface deformation, and the key machining parameters.

**Figure 9:**
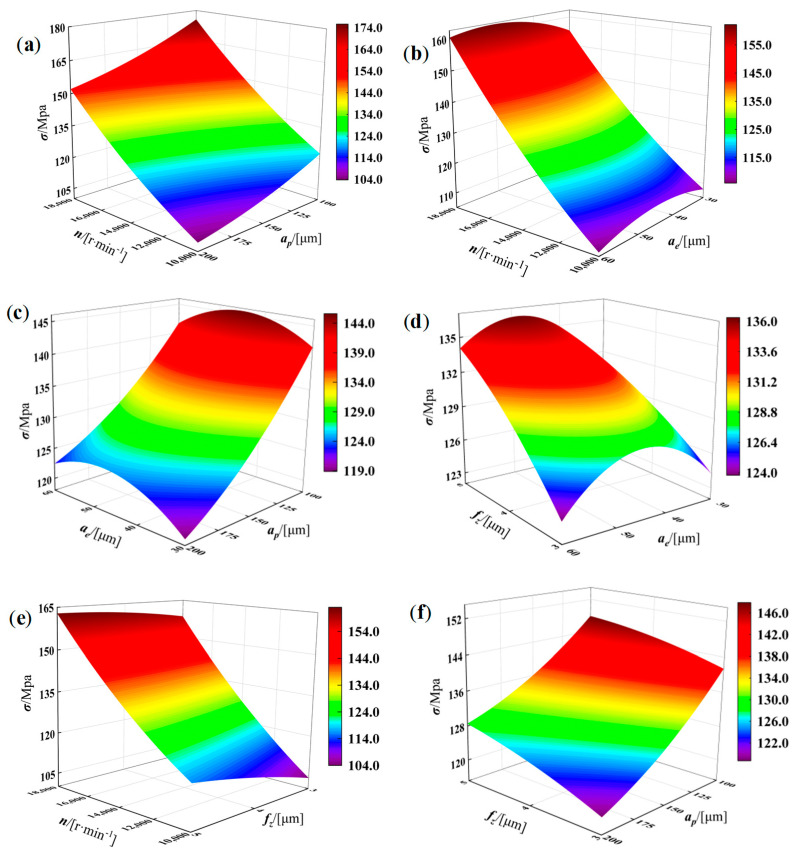
Effects of micro−milling parameters on the residual stress *σ* of curved micro−thin−walled structures. (**a**) *n* and *a_p_* (**b**) *n* and *a_e_* (**c**) *a_e_* and *a_p_* (**d**) *f_z_* and *a_e_* (**e**) *n* and *f_z_* (**f**) *f_z_* and *a_p_*.

**Figure 10:**
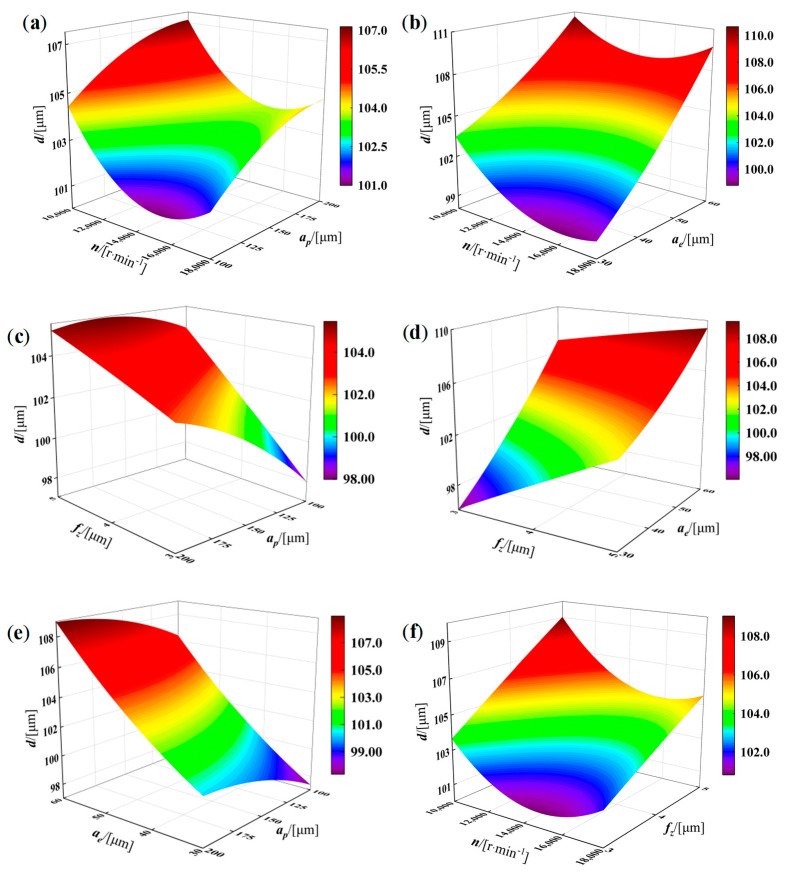
Effects of micro−milling parameters on the machining deformation *d* of curved micro−thin−walled. (**a**) *n* and *a_p_* (**b**) *n* and *a_e_* (**c**) *f_z_* and *a_p_* (**d**) f*_z_* and *a_e_* (**e**) *a_e_* and *a_p_* (**f**) *n* and *f_z_*.

The response surface plots shown in Figure 9 and Figure 10 illustrate the overall trends of residual stress and top-surface deformation as influenced by the machining parameters. By jointly examining Figure 9 and Figure 10 together with the corresponding ANOVA results, it is evident that both the individual parameters and their interactions exert significant effects on the residual stress. As indicated in Figure 9, decreasing *n* and *f_x_* can markedly reduce the magnitude of residual stress, whereas a smaller axial depth of cut *a_p_* (100~200 μm) tends to increase it. Along the radial depth-of-cut direction, the residual stress presents a characteristic “low~high~low” distribution; therefore, when selecting relatively large *n* and *a_p_*, a smaller or larger *a_e_* (30~60 μm) should be employed to mitigate the adverse influence of increased material removal and consequently obtain lower residual stress. Likewise, Figure 10 shows that top-surface deformation is more sensitive to variations in *n* and *a_p_*; decreasing *f_x_* and *a_e_* can significantly reduce deformation. As *n* increases within the range of (10,000~18,000 r/min), the deformation first decreases and then increases, and with increasing *a_p_*, the deformation also displays a “low~high~low” pattern. Therefore, when choosing relatively large *f_x_* and *a_e_*, a smaller or larger *a_p_* should be selected to counteract the unfavorable effects of excessive cutting engagement and ultimately achieve minimal deformation.

## 4. Establishment of a Micro-Milling-Induced Residual Stress and Deformation Prediction Model and Its Objective Optimization

### 4.1. GA–BP Neural Network Prediction Models

The classical architecture of a Backpropagation (BP) neural network typically consists of three components: an input layer, a hidden layer, and an output layer, each comprising numerous neurons. During forward propagation, the network produces the predictive output, whereas during back-propagation, the initial weights and thresholds are updated according to gradient descent on the loss function. As the fundamental information-processing units, neurons interact and transmit information following specific organizational rules. To achieve superior predictive performance, the number of neurons (*L*) in the hidden layer is commonly determined using Formula (9).

(9) $$L=\sqrt{n+m}+a$$

where *n* denotes the number of input units, *m* represents the number of output units, and *a* is a constant within the range [1,10].

**Figure 11:**
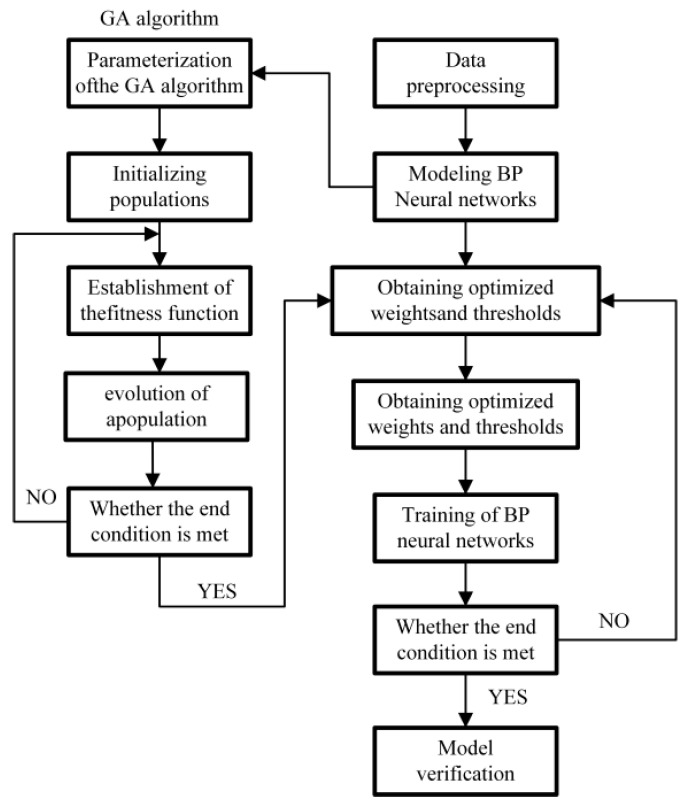
Algorithmic flowchart of the GA-BP neural network.

**Figure 12:**
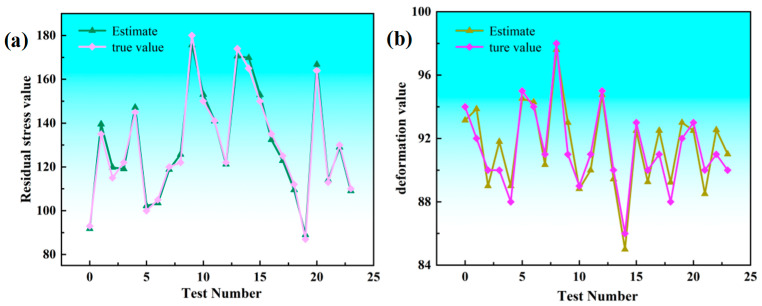
Comparison between the actual and predicted target values: (**a**) residual stress value; (**b**) deformation value.

The BP neural network is prone to converging to local optima, and insufficiently comprehensive samples may lead to overfitting during training. To address these limitations, a population-based intelligent optimization algorithm is integrated with the BP neural network to improve its performance. The Genetic Algorithm (GA), inspired by the biological evolutionary process, has been widely applied to solving both constrained and unconstrained optimization problems. By combining GA with the BP neural network, a GA-BP neural network is proposed, where GA is employed to optimize the network’s initial weights and thresholds, after which the refined search space is further tuned using BP.GA is employed to optimize the network’s initial weights and thresholds, after which the refined search space is further tuned using BP. The algorithmic workflow of the GA-BP neural network is illustrated in Figure 11. In this study, the population size, maximum number of iterations, crossover probability, and mutation probability are set to 60, 200, 0.4, and 0.1, respectively. The GA-BP neural network is then trained, and a comparison between the predicted and actual values is presented in Figure 12, demonstrating that the GA-BP-based residual stress prediction model achieves high prediction accuracy. The high-precision prediction results of the GA-BP neural network provide reliable input for the subsequent multi-objective optimization, as accurate prediction of residual stress and deformation is a prerequisite for identifying optimal machining parameters that simultaneously minimize both responses. To quantify the predictive performance of the model, the core evaluation indicators of the GA-BP neural network are summarized in Table 4.

### 4.2. AGE-MOEA2 Algorithm

Building on the GA-BP-based prediction model, this section adopts the AGE-MOEA2 algorithm to optimize the four key micro-milling parameters (*n*, *f*_z_, *a*_e_, *a*_p_). The optimization objective is to minimize residual stress and top-surface deformation. The AGE-MOEA2 algorithm is selected for its superior performance in handling complex multi-objective optimization problems, especially in balancing convergence and diversity of Pareto-optimal solutions. AGE-MOEA2 is a multi-objective optimization algorithm developed on the basis of a grid-partitioning strategy and serves as an extension and refinement of the MOEAD framework for solving complex multi-objective optimization problems. Considering that surface residual stress has a pronounced influence on the deformation behavior of low-rigidity thin-walled components, minimizing the residual stress induced during micro-milling is essential for reducing deformation. Accordingly, to obtain the optimal machining parameters corresponding to the minimum residual stress *σ* and top surface deformation *d*, *σ* and *d* are defined as the objective functions, while spindle speed *n*, feed per tooth *f_z_*, axial depth of cut *a_p_*, and radial depth of cut *a_e_* are designated as the decision variables. On this basis, a multi-objective optimization model is established as expressed in Formula (10).

(10) $$\begin{array}{l} find:x_{1},x_{2},x_{3},x_{4}\\ min:\left\{\begin{array}{l} f_{1}(x_{1},x_{2},x_{3},x_{4})=d\\ f_{2}(x_{1},x_{2},x_{3},x_{4})=\sigma \end{array}\right.\\ s.t.:\left\{\begin{array}{l} 10000\leq x_{1}\leq 18000\\ 3\leq x_{2}\leq 5\\ 30\leq x_{3}\leq 60\\ 100\leq x_{4}\leq 300 \end{array}\right. \end{array}$$

where *d* denotes the thickness of the micro-thin wall, *σ* represents the residual stress, and *x*_1_*~x*_4_ correspond to the machining parameters to be optimized, namely the spindle speed *n*, feed per tooth *f_z_*, radial depth of cut *a_e_*, and axial depth of cut *a_p_*, respectively.

**Figure 13:**
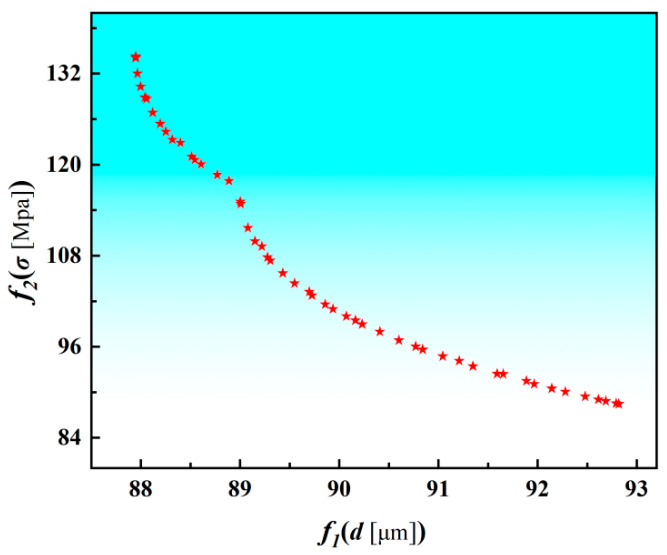
Pareto−optimal solution set.

**Figure 14:**
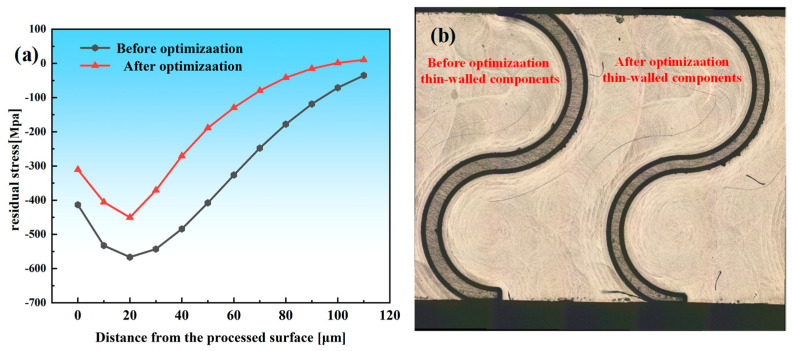
Comparison chart of micro-milling parameters before and after optimization. (**a**) Residual stress distribution curve. (**b**) Deformation diagram of micro−milling thin-walled parts.

According to the multi-objective optimization model for the micro-milling parameters, the maximum number of iterations is set to *N* = 800 and the population size to *p* = 350. Figure 13 illustrates the evolution of the Pareto front under different iteration counts. Based on the optimization results, machining parameters of *n* = 15,840.82 r/min, *f_z_* = 3.03 μm/z, *a_e_* = 42.32 μm, and *a_p_* = 300.00 μm were applied to conduct validation experiments on the micro thin-wall structure. After optimization, the trends of the maximum compressive residual stress along the depth direction and the equivalent in-plane residual stress amplitude remained consistent, indicating that the overall residual stress field was effectively reduced. The measured residual stress distribution and the corresponding thin-wall deformation are shown in Figure 14. It can be observed that the optimized parameters lead to a pronounced decrease in the peak compressive residual stress, while the wall thickness becomes more uniform and the deformation is simultaneously reduced.

**Figure 15:**
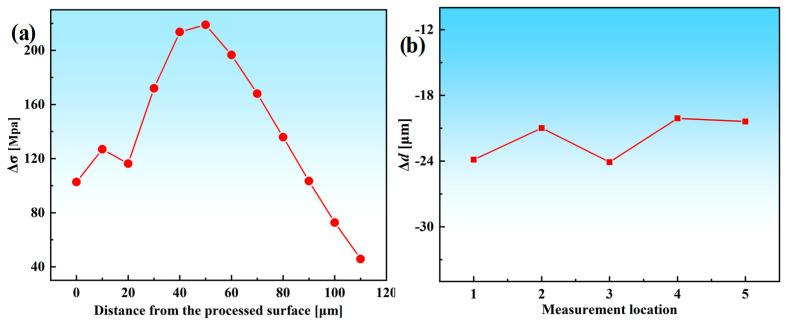
Comparison of micro−milling results before and after optimization. (**a**) Improvement curve of residual stress (Δ*σ*). (**b**) Deformation reduction at five measurement locations (Δ*d*).

**Figure 16:**
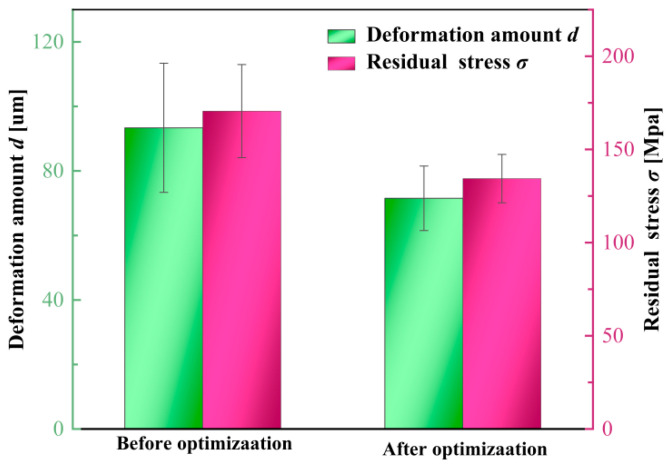
Statistical analysis of residual stress and top-surface deformation errors before and after precision optimization (the error bars represent the 95% confidence intervals).

To quantitatively compare the variation magnitude and consistency across different depth segments and measurement locations, the improvement in residual stress along the depth direction is expressed as Δ*σ*, and the reduction in top-surface deformation at the *i*-th measurement point (*i* = 1~5) is expressed as Δ*d*. As shown in Figure 15a, Δ*σ* remains positive throughout the entire depth range, indicating that the optimized parameters effectively mitigate the original compressive residual stress peaks. A pronounced improvement appears in the 30~60 μm region, suggesting that the optimized cutting parameters significantly alleviate stress concentration within the surface and subsurface layers. Figure 15b illustrates the deformation reduction at five measurement points of the thin-walled structure. The clustered distribution of Δ*d* values indicates that the optimized parameters not only reduce the overall deformation but also substantially enhance deformation uniformity along the machining direction, preventing localized distortion. Quantitative comparison of the pre- and post-optimization results shows that the residual stress is reduced by 21.233% and the thin-wall deformation by 23.412%, as presented in Figure 16. Comprehensive analysis of Figure 14, Figure 15 and Figure 16 demonstrates that the optimized multi-objective parameters effectively attenuate the compressive residual stress peaks while achieving a significant reduction in the overall deformation of the thin-walled component.

## 5. Conclusions

In this study, a comprehensive framework integrating theoretical modeling, experimental analysis, and multi-objective optimization was developed to address the challenges of residual stress accumulation, machining deformation, and dimensional accuracy in the micro-milling of TC4 titanium alloy thin-walled components for aerospace applications. An exponential-decay sinusoidal model was first established to characterize the subsurface residual stress distribution, and an improved particle swarm algorithm was employed to achieve high-accuracy parameter identification. Subsequently, high-speed micro-milling experiments based on response surface methodology were conducted to quantify the effects of spindle speed, feed per tooth, radial depth of cut, and axial depth of cut on both residual stress and top-surface deformation, thereby providing a predictive basis for process parameter matching. To further enhance prediction capability under complex machining conditions, a GA-BP neural network model was constructed, effectively overcoming the local-minimum limitations of conventional BP networks and improving prediction accuracy for both residual stress and deformation responses. Building upon these models, a multi-objective optimization strategy targeting the simultaneous minimization of residual stress and deformation was formulated using the AGE-MOEA2 algorithm. Experimental validation using the optimized parameters demonstrated a 23.412% reduction in top-surface deformation and a 21.233% decrease in residual stress compared with unoptimized conditions, confirming the robustness and effectiveness of the proposed integrated methodology in improving machining precision and structural stability of curved thin-walled TC4 components.

## Figures and Tables

**Table 1:** Surface residual stress measurement values.

Machining Parameters		Distance of the Measurement Point from the Machined Surface [μm]							
*f*_z_	*v*_c_	0	10	20	30	50	70	90	110
mm/z	m/min	Residual stress value [MPa]							
0.03	45	−415	−525	−595	−520	−390	−295	−145	70
0.05	45	−540	−695	−755	−670	−455	−350	−275	5
0.04	30	−440	−535	−535	−495	−470	−125	−10	20
0.04	60	−15	−115	−130	−205	−115	−28	10	−18
0.03	60	−75	−105	−135	−155	−55	12	22	18
0.05	30	−445	−615	−560	−445	−305	−45	28	15
0.03	30	−205	−245	−345	−325	−195	−85	45	−25
0.04	45	−475	−590	−640	−705	−585	−295	−270	85
0.05	60	−105	−125	−188	−270	−140	−52	−18	12

**Table 2:** Characterization coefficients of the residual stress distribution curves.

*f*_z_	*v*_c_	ζ	λ	κ	φ	*R* ^2^
mm/z	m/min					
0.03	45	176.09	0.99	0.022480	3.4792	0.95744
0.05	45	201.09	0.99	0.020157	3.5531	0.95061
0.04	30	706.35	0.57	0.027249	3.6745	0.96848
0.04	60	100.00	0.98	0.031889	3.1511	0.91421
0.03	60	206.79	0.50	0.041380	3.4157	0.96777
0.05	30	180.51	0.99	0.031432	3.5034	0.98738
0.03	30	116.04	0.99	0.029560	3.3618	0.94774
0.04	45	231.45	0.99	0.022098	3.4140	0.93583
0.05	60	318.99	0.68	0.030799	3.3327	0.91809

**Table 3:** Experimental design table.

Standard	Run	*n*	*f_z_*	*a_e_*	*a_p_*	*d*	*σ*
Sequence	Sequence	r/min	μm/z	μm	μm	μm	Mpa
13	1	10,000	3	60	300	93.144	91.7
14	2	18,000	3	60	300	93.859	139.4
24	3	14,000	4	45	300	89.523	119.8
21	4	14,000	4	30	200	91.821	119.0
23	5	14,000	4	45	100	89.156	147.1
11	6	10,000	5	30	300	94.518	102.2
17	7	10,000	4	45	200	94.327	103.5
19	8	14,000	3	45	200	90.337	118.8
3	9	10,000	5	30	100	97.618	125.7
8	10	18,000	5	60	100	93.238	175.6
16	11	18,000	5	60	300	88.816	153.1
12	12	18,000	5	30	300	90.428	140.9
7	13	10,000	5	60	100	94.748	121.2
6	14	18,000	3	60	100	89.435	170.5
2	15	18,000	3	30	100	85.267	169.8
18	16	18,000	4	45	200	92.525	152.8
10	17	18,000	3	30	300	89.254	132.4
22	18	14,000	4	60	200	92.547	122.8
5	19	10,000	3	60	100	89.228	109.4
9	20	10,000	3	30	300	93.168	89.1
4	21	18,000	5	30	100	92.542	166.8
1	22	10,000	3	30	100	88.531	114.0
20	23	14,000	5	45	200	92.544	128.9
15	24	10,000	5	60	300	91.009	109.1

**Table 4:** Core evaluation indicators of the GA-BP neural network model.

	MSE	RMSE	MAE	R^2^
Residual stress	7.7419	2.7824	2.4510	0.9622
Top-surface deformation	1.1764	1.0846	0.9537	0.9473

## Data Availability

The original contributions presented in the study are included in the article; further inquiries can be directed to the corresponding author.
